# Supplementary material for: Chromatin organization in the female mouse brain fluctuates across the oestrous cycle
Source: Nat Commun. 2019 Jun 28;10:2851. doi: 10.1038/s41467-019-10704-0 (PMC6598989; doi:10.1038/s41467-019-10704-0)
Supplement: Supplementary file 1 — Supplementary Information [file 41467_2019_10704_MOESM1_ESM.pdf]

Supplementary Information

Chromatin organization in the female mouse brain fluctuates across the oestrous cycle

Jaric et al.

Table of Contents

I. Supplementary Figures:

Supplementary Figure 1. Oestrous cycle stage determination and sex hormone levels

Supplementary Figure 2. Oestrous cycle- and sex-dependent regulation of *Ppp1r1b* chromatin organization

Supplementary Figure 3. Model of oestrogen-induced chromatin organizational changes in the ventral hippocampus during proestrus

Supplementary Figure 4. Co-expression of oestrogen receptor β (ERβ), Darpp32, and neuronal marker MAP2 in the ventral hippocampus

Supplementary Figure 5. Separation of neuronal nuclei using fluorescence-activated nuclei sorting (FANS)

Supplementary Figure 6. ATAC-seq library quality control

Supplementary Figure 7. nucRNA and cDNA library quality control

II. Supplementary Tables:

Supplementary Table 1: ATAC-seq data basic information

Supplementary Table 2: nucRNA-seq data basic information

Supplementary Figure 1

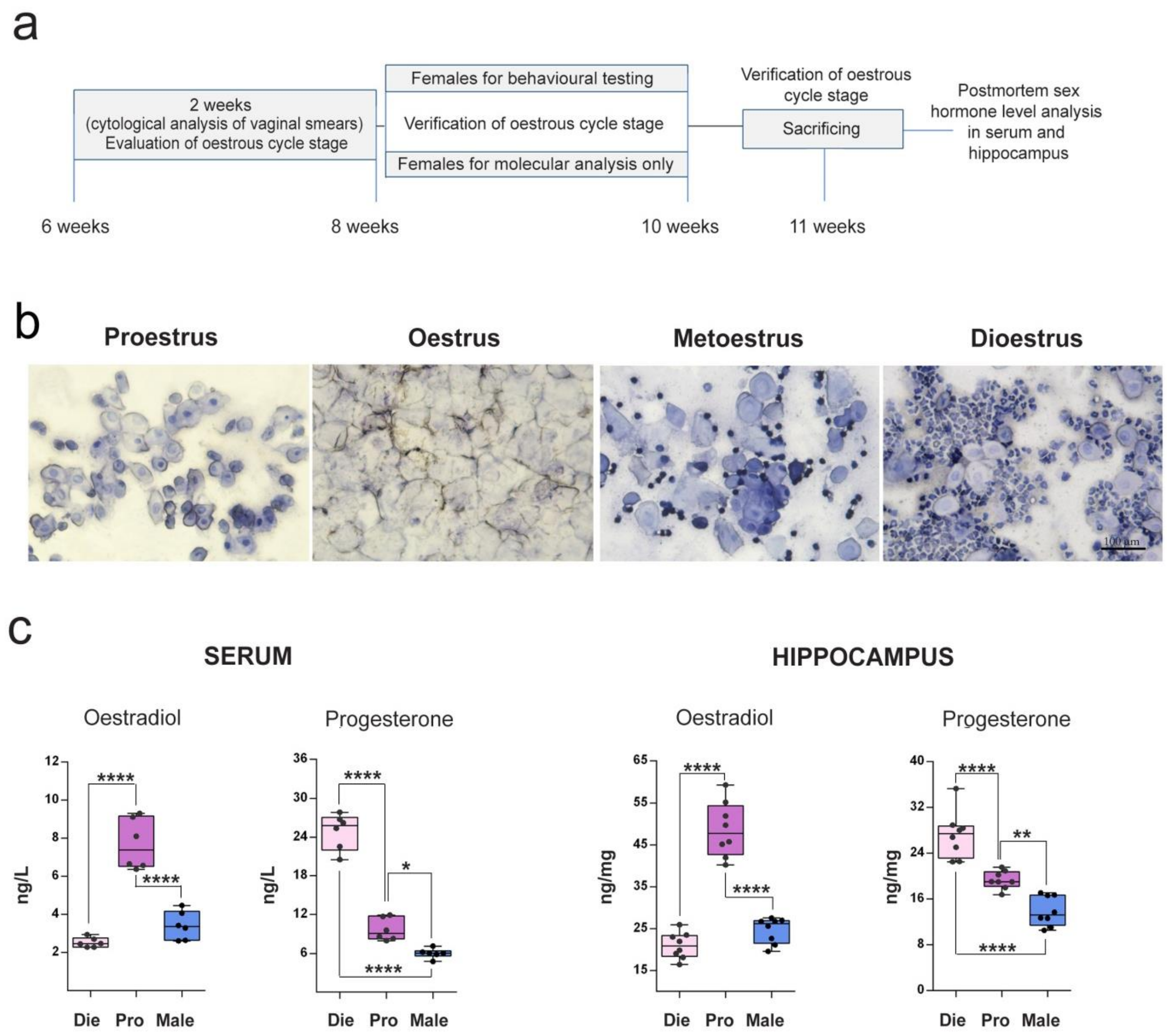

Oestrous cycle stage determination and sex hormone levels. a, The oestrous cycle patterns were checked daily, from 6-8 weeks of age, using the vaginal cytological analysis. From 8-10 weeks, the stage was confirmed at the time of testing (in behaviourally tested animals) and, simultaneously, in their littermates used for molecular analysis. One cycle following the last test, animals were sacrificed, the cycle stage was verified again, and serum and the hippocampi were isolated for the analysis of sex hormone levels using ELISA. **b,** The mouse oestrous cycle typically lasts 4-5 days and consists of proestrus, oestrus, metoestrus, and dioestrus. The cycle stage was determined based on the cell types present in vaginal smear. In proestrus, mostly round, nucleated epithelial cells and some cornified epithelial cells are present. At oestrus, mostly cornified epithelial cells are seen. Metoestrus is associated with cornified eipithelial cells and polymorphonuclear leukocytes and some nucleated epithelial cells. Dioestrus shows primarily polymorphonuclear leukocytes and a few epithelial cells during late dioestrus. **c,** ELISA tests measured oestradiol and progesterone levels in serum and the hippocampus *postmortem* and confirmed that sex hormone levels corresponded to the cycle stage as determined by the cytological analysis. Note high oestrogen-low progesterone levels in proestrus females; low oestrogen-high progesterone levels in dioestrus females; and low oestrogen-low progesterone levels in males. The levels in serum and the hippocampus were concordant.

Supplementary Figure 2

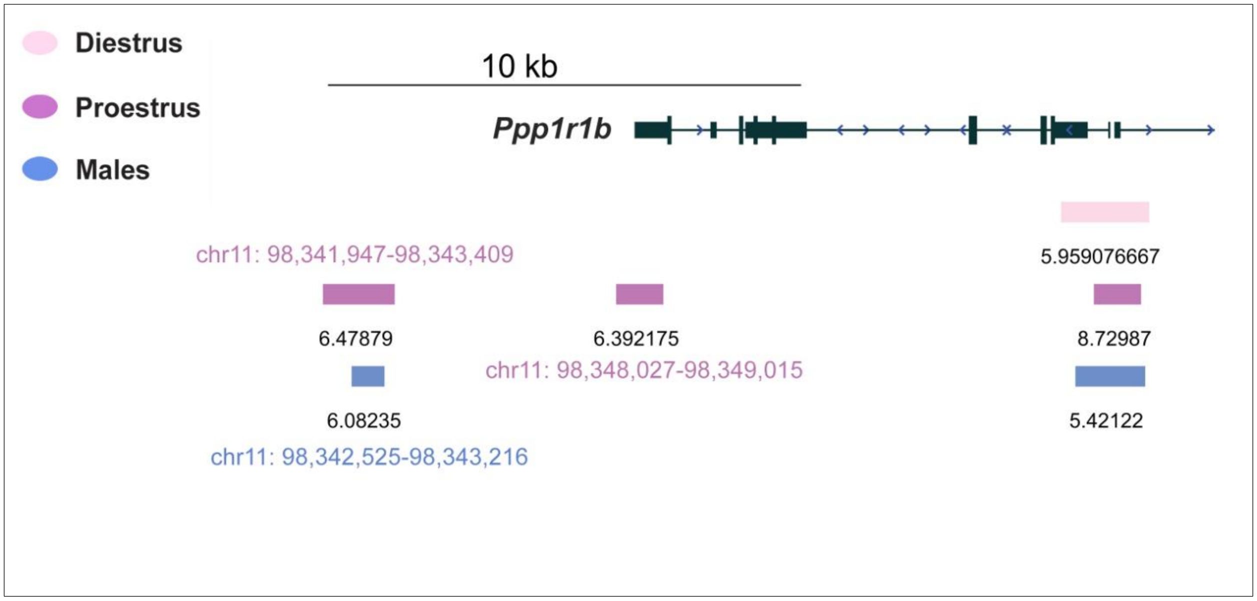

Oestrous cycle- and sex-dependent regulation of *Ppp1r1b* chromatin organization. The figure shows the location of the ATAC-seq peaks in the vicinity of the *Ppp1r1b* gene in ventral hippocampal neurons isolated from dioestrus females, proestrus females, and males. The figure is generated in the IGV using the merged bed files for each group. Note that the peaks are oestrous cycle- and sex-specific.

Supplementary Figure 3

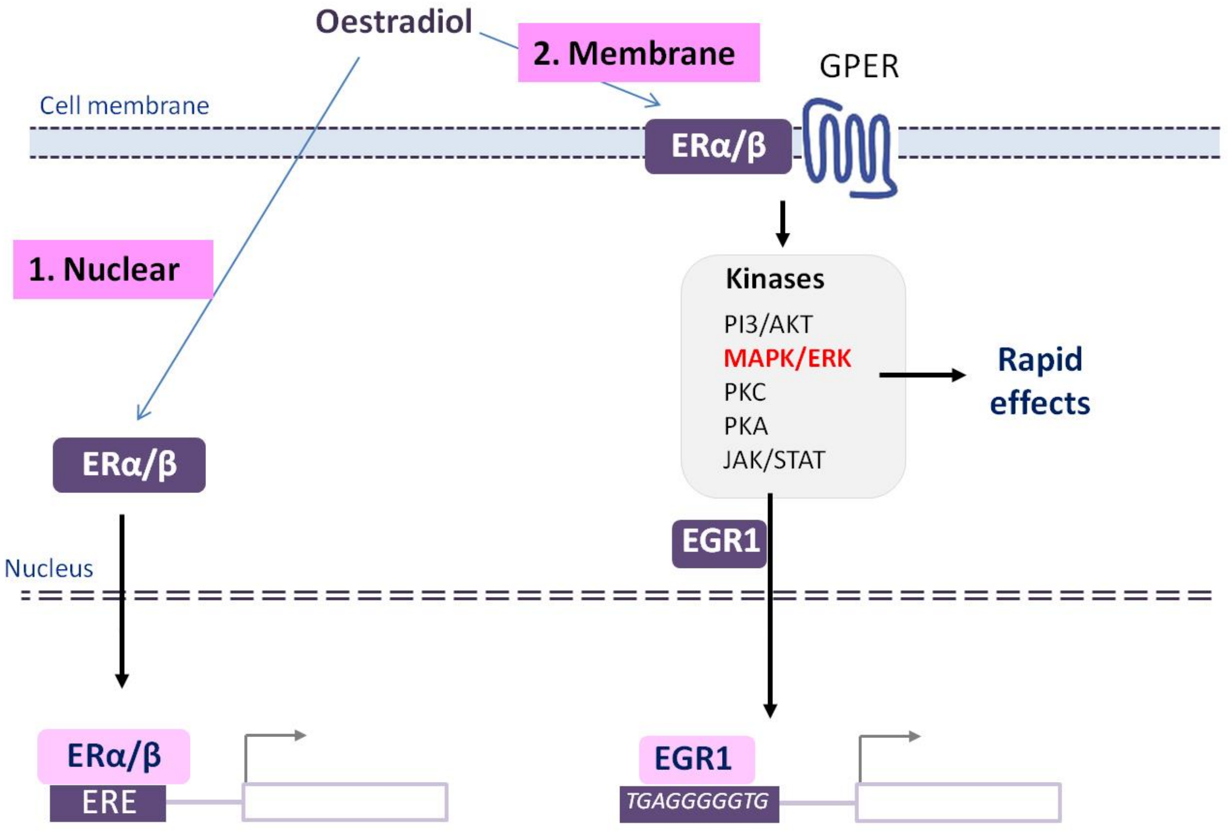

Model of oestrogen-induced chromatin organizational changes in the ventral hippocampus during proestrus. High oestradiol levels during proestrus can induce genomic effects through a nuclear receptor-dependent mechanism (1) or via a membrane receptor-dependent mechanism (2). Direct genomic effects are exerted through classical, nuclear oestrogen receptors (ERα or ERβ) which, upon the activation by oestradiol, bind oestrogen response elements (ERE) in the DNA and activate transcription (left). On the contrary, binding of oestradiol to the membrane oestrogen receptors (ERα, ERβ, and G-protein coupled ER, GPER) can activate multiple kinase pathways, including the MAPK/ERK pathway, which, in turn, induce phosphorylation of numerous cellular proteins leading to rapid cellular effects or indirect genomic effects (right). Our data support the membrane receptor-associated model (right) in which oestrogen induces the expression of an immediate early gene, Egr1, resulting in increased chromatin accessibility of Egr1 motif-containing loci and increased expression of nearby, Egr1-regulated genes. It is likely that, during proestrus, Mef2 transcription factors also gain increased access to their DNA binding sites in ventral hippocampal neurons via a membrane-receptor associated mechanism.

Supplementary Figure 4

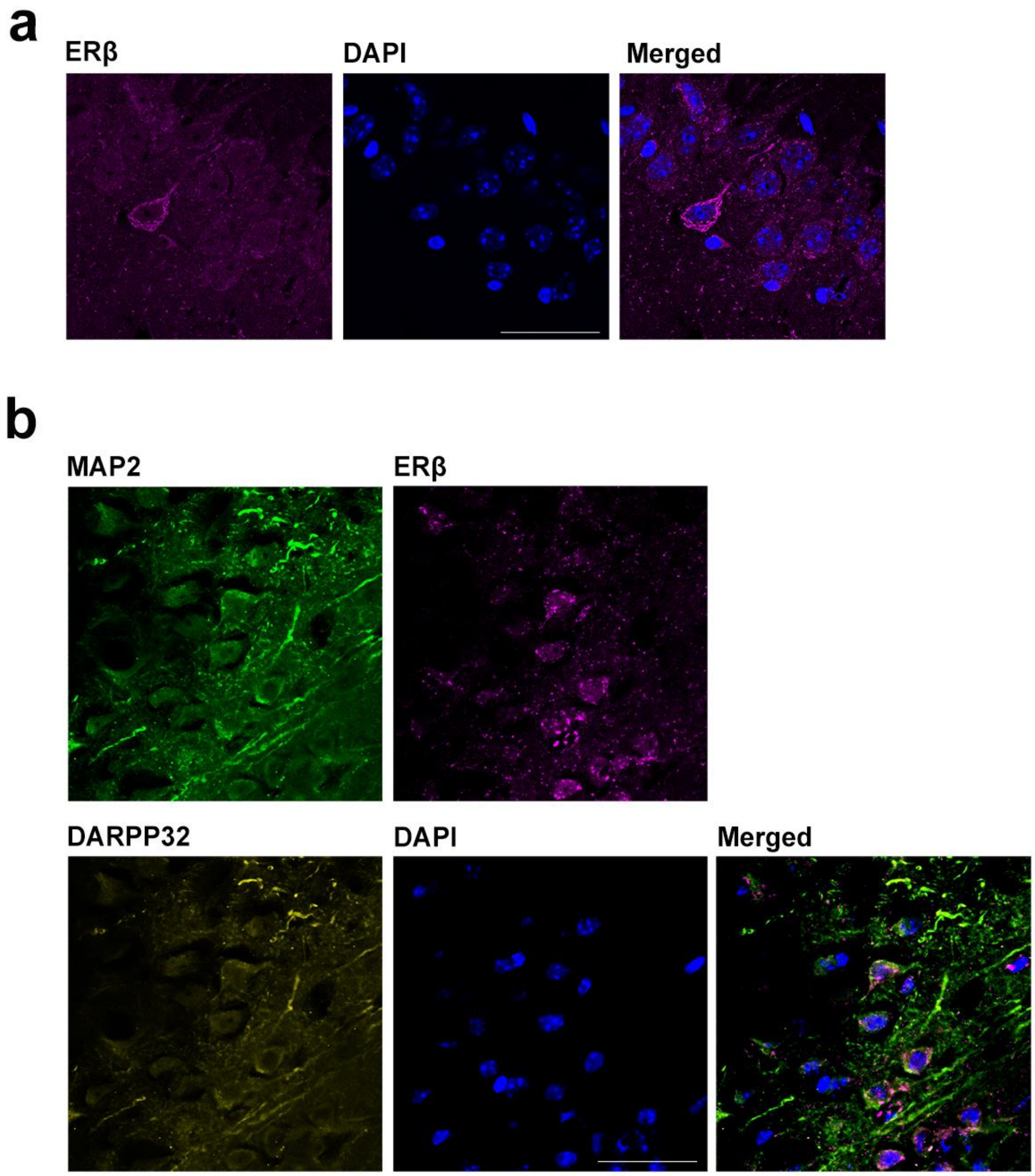

Co-expression of oestrogen receptor β (ERβ), Darpp32, and neuronal marker MAP2 in the ventral hippocampus a. Confocal images show that ERβ is primarily located in the membrane and cytosol with limited nuclear localization in the ventral hippocampus. **b,** We also confirm that ERβ is co-expressed with our candidate protein Darpp32 in hippocampal neurons (containing MAP2 marker).

Supplementary Figure 5

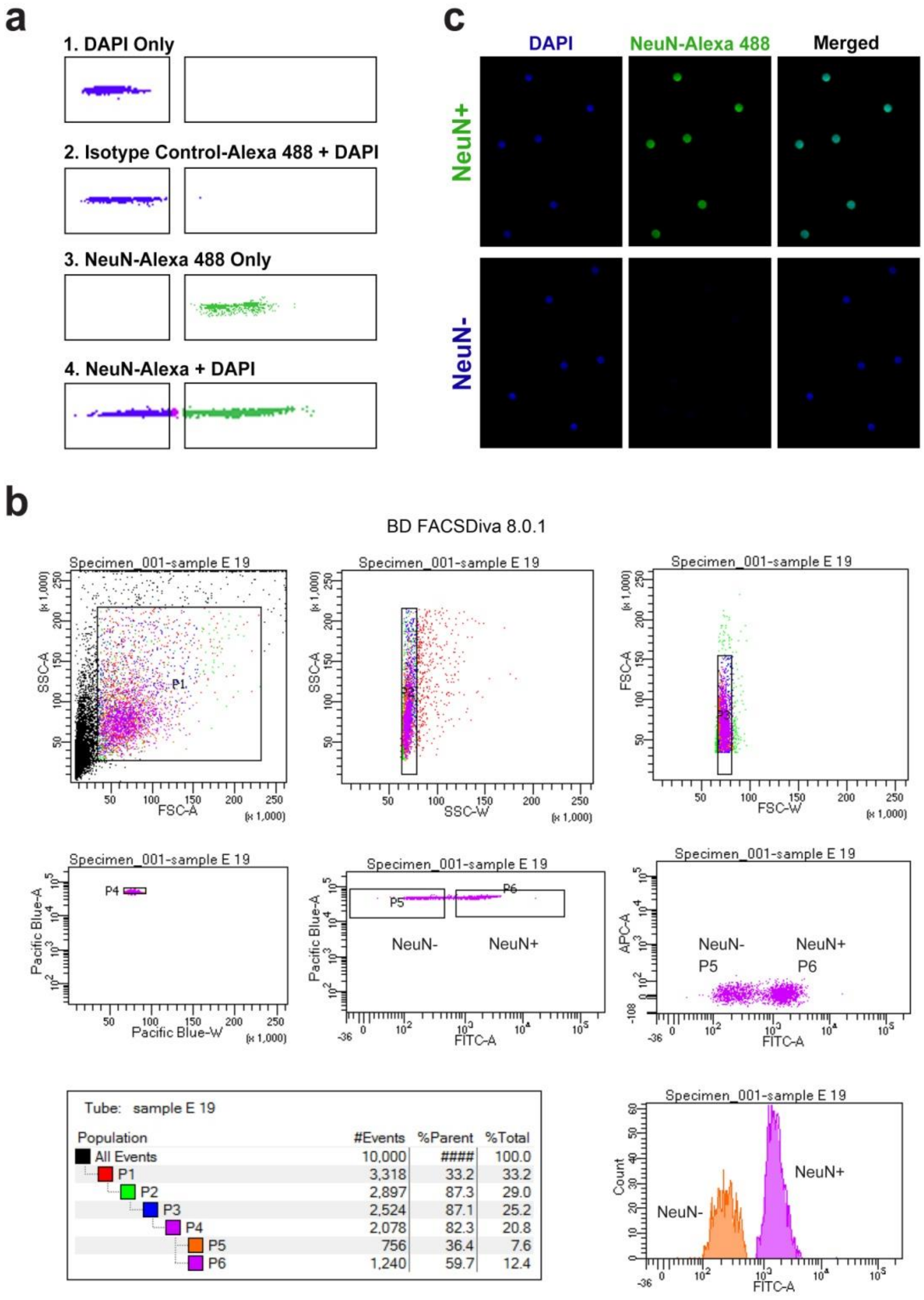

Separation of neuronal nuclei using fluorescence-activated nuclei sorting (FANS). a. Sorting plots from: two negative controls - DAPI only (1) and isotype control + DAPI (2) -processed without NeuN antibody; positive control containing NeuN antibody only (3), and our sample processed with NeuN antibody and DAPI (4). b) Representative FANS report showing the gating strategy for the removal of debris and ensuring a successful separation of NeuN+ (neuronal) and NeuN- (non-neuronal) single nuclei. c) Fluorescence micrographs show NeuN/DAPI staining and larger size of the sorted neuronal (NeuN+) nuclei (upper panel), and DAPI only staining and smaller size of the sorted non-neuronal (NeuN-) nuclei (lower panel).

Supplementary Figure 6

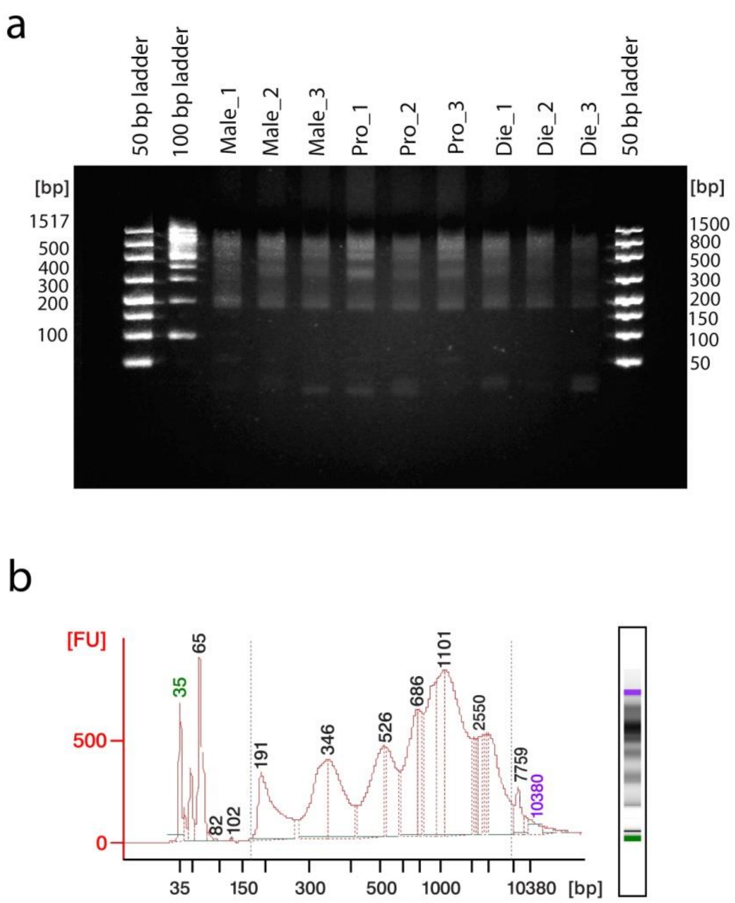

ATAC-seq library quality control. The quality control of our ATAC-seq libraries was performed using (a) agarose gel electophoresis and (b) Bioanalyzer. The representative gel and Bioanalyzer trace are shown. Note a clear, nucleosomal banding pattern in both images, typical of high-quality libraries.

Supplementary Figure 7

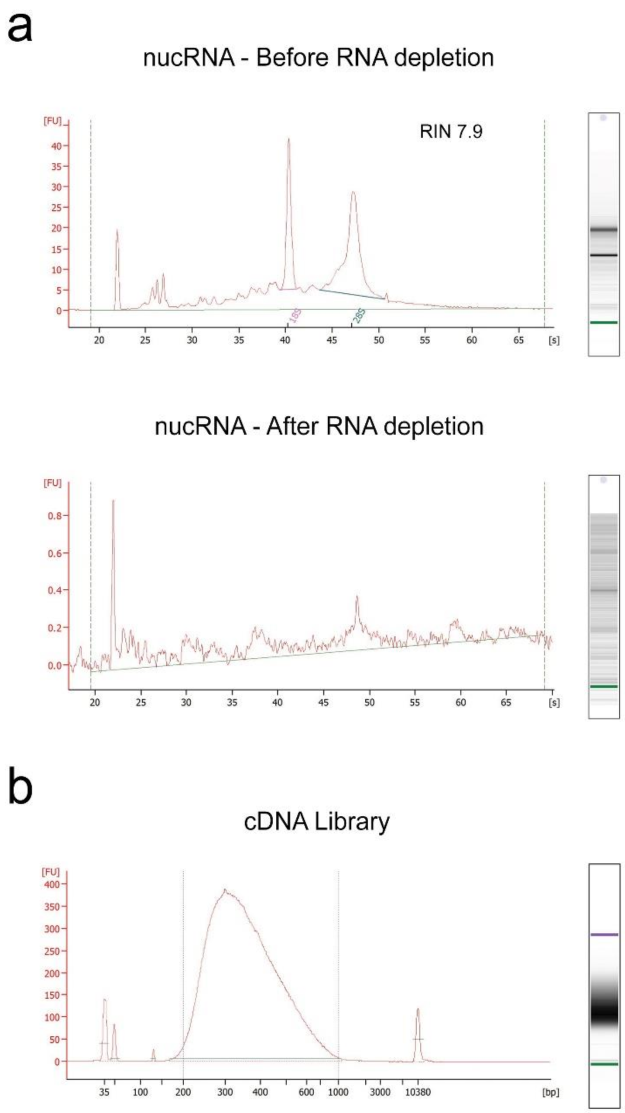

nucRNA and cDNA library quality control. a) Bioanalyzer trace of a representative nucRNA sample before and after rRNA depletion. b) Bioanalyzer trace of a representative cDNA library.

Supplementary Table 1. ATAC-seq data basic information

Sample ID	Sex	Condition	Replicate	Original read number	% mapped	% uniquely aligned	% chrM	% duplicate	Read length/paired- or single-end
Pro_1	Female	Proestrus	1	117,360,623	97.18%	87.01%	0.65%	3.95%	100 bp-paired end
Pro_2	Female	Proestrus	2	119,794,451	88.17%	73.62%	0.78%	8.56%	100 bp-paired end
Pro_3	Female	Proestrus	3	113,562,161	98.11%	88.88%	0.76%	3.30%	100 bp-paired end
Die_1	Female	Dioestrus	1	101,456,853	89.20%	82.87%	0.96%	5.35%	100 bp-paired end
Die_2	Female	Dioestrus	2	86,181,020	96.97%	90.38%	0.60%	2.88%	100 bp-paired end
Die_3	Female	Dioestrus	3	108,772,328	97.60%	87.34%	1.58%	4.48%	100 bp-paired end
Male_1	Male	Males	1	97,035,861	96.88%	85.95%	0.82%	3.52%	100 bp-paired end
Male_2	Male	Males	2	98,966,730	81.49%	70.82%	1.08%	6.51%	100 bp-paired end
Male_3	Male	Males	3	132,315,424	98.30%	87.61%	0.91%	3.77%	100 bp-paired end

Sample ID	Chromosome 1					Down-sampling		Detected peak number in genome-wide (final)
	Chromosome 1 reads	Filt % *	FragL†	RiP % ‡	Estimated reads in peak	Fraction mean	Down-sampling factor	
Pro_1	8,600,258	0.665	206	22.6	1943658.3	78.9	0.79	180,589
Pro_2	7,572,455	1.11	225	13.5	1022281.4	150	1	236,138
Pro_3	8,187,984	0.608	214	25.2	2063372	74.3	0.74	187,917
Die_1	6,708,404	0.861	216	20.6	1381931.2	111	1	248,157
Die_2	6,488,236	0.727	215	20	1297647.2	118.2	1	214,760
Die_3	7,054,570	0.664	188	21.9	1544950.8	99.3	0.99	310,212
Male_1	6,822,446	0.699	185	14.6	996077.1	154	1	279,794
Male_2	6,201,168	0.759	215	22.8	1413866.3	108.5	1	181,541
Male_3	9,734,317	0.618	217	22	2141549.7	71.6	0.72	227,646

* **Filt%,** Percentage of mapped reads passing MapQ filter,

†**FragL,** Predicted fragment length by cross-coverage method

‡**RiP%,** Reads mapped to peaks

Supplementary Table 2. nucRNA-seq data basic information

**Sample name**	**NRNA 1 _CGATGT**	**NRNA-2 _CAGATC**	**NRNA-3 _GTGAAACG**	**NRNA-4 _ACAGTG**	**NRNA-5 _GCCAAT**	**NRNA-6 _ATGTCAGA**	**NRNA-7 _TGACCA**	**NRNA-8 _CTTGTA**	**NRNA-9 _CCGTCCCG**
Sex	Female	Female	Males	Female	Female	Males	Female	Female	Males
Status	Proestrus	Dioestrus	Males	Proestrus	Dioestrus	Males	Proestrus	Dioestrus	Males
Replicate	1	1	1	2	2	2	3	3	3
Number of input reads	40644745	42986843	39132784	38965232	41083495	42708908	40857646	41990435	41930741
Average input read length	197	197	196	197	196	197	196	196	196
**UNIQUE READS**									
Uniquely mapped reads number	36951997	39551655	35682821	35333284	37381559	39356713	37031803	38031809	38479560
Uniquely mapped reads%	90.91%	92.01%	91.18%	90.68%	90.99%	92.15%	90.64%	90.57%	91.77%
Average mapped length	196.47	197.1	196.38	196.77	196.4	196.62	195.91	196.23	196.23
Number of splices: Total	7654719	6864657	7175575	8087137	9411656	6226993	9137060	9467422	7193929
Number of splices: Annotated (sjdb)	7545655	6754151	7068345	7979031	9293892	6122032	9018137	9345859	7088018
Number of splices:GT/AG	7583600	6800981	7107676	8010447	9322801	6169244	9050914	9377681	7125569
Number of splices: GC/AG	60100	53678	57032	64979	75341	48515	72926	75830	57794
Number of splices: AT/AC	7489	6853	7489	7976	9482	6265	9106	9550	7292
Number of splices: Non-canonical	3530	3145	3378	3735	4032	2969	4114	4361	3274
Mismatch rate per base, %	0.23%	0.21%	0.23%	0.23%	0.22%	0.23%	0.22%	0.22%	0.22%
Deletion rate per base	0.01%	0.01%	0.02%	0.02%	0.01%	0.01%	0.01%	0.02%	0.01%
Deletion average length	2.49	2.4	2.62	2.52	2.37	2.31	2.56	2.59	2.43
Insertion rate per base	0.01%	0.01%	0.01%	0.01%	0.01%	0.01%	0.01%	0.01%	0.01%
Insertion average length	1.62	1.54	1.59	1.62	1.53	1.49	1.59	1.6	1.52

**MULTI-MAPPING READS**									
Number of reads mapped to multiple loci	1147420	1056879	1113360	1255050	1346594	1042547	1361986	1315460	1067063
% of reads mapped to multiple loci	2.82%	2.46%	2.85%	3.22%	3.28%	2.44%	3.33%	3.13%	2.54%
Number of reads mapped to too many loci	85036	92310	88496	81406	83085	88963	100103	94075	93202
% of reads mapped to too many loci	0.21%	0.21%	0.23%	0.21%	0.20%	0.21%	0.25%	0.22%	0.22%
**UNMAPPED READS**									
% of reads unmapped: too many mismatches	0.00%	0.00%	0.00%	0.00%	0.00%	0.00%	0.00%	0.00%	0.00%
% of reads unmapped: too short	5.14%	4.27%	4.73%	5.03%	4.73%	4.12%	4.82%	5.23%	4.48%
% of reads unmapped: other	0.91%	1.05%	1.01%	0.86%	0.80%	1.08%	0.96%	0.84%	0.98%
**CHIMERIC READS**									
Number of chimeric reads	0	0	0	0	0	0	0	0	0
% of chimeric reads	0.00%	0.00%	0.00%	0.00%	0.00%	0.00%	0.00%	0.00%	0.00%
