# Supplementary material for: Chromatin organization in the female mouse brain fluctuates across the oestrous cycle
Source: Nat Commun. 2019 Jun 28;10:2851. doi: 10.1038/s41467-019-10704-0 (PMC6598989; doi:10.1038/s41467-019-10704-0)
Supplement: Supplementary file 3 — Description of Additional Supplementary Files [file 41467_2019_10704_MOESM3_ESM.pdf]

Description of Additional Supplementary Information

File name: Supplementary Data 1
Description: Differential ATAC-seq peaks

File name: Supplementary Data 2
Description: Significantly enriched GO terms in genes associated with differential chromatin accessibility

File name: Supplementary Data 3
Description: Significantly enriched KEGG pathways in genes associated with differential chromatin accessibility

File name: Supplementary Data 4
Description: Homer motif analysis of ATAC-seq data

File name: Supplementary Data 5
Description: KEGG pathways enriched in proestrus-specific, Egr1 motif-containing genes (ATAC-seq, dioestrus-proestrus comparison)

File name: Supplementary Data 6
Description: nucRNA-seq Differentially Expressed Genes

File name: Supplementary Data 7
Description: Significantly enriched GO terms in nucRNA-seq Differentially Expressed Genes

File name: Supplementary Data 8
Description: Significantly enriched KEGG pathways in nucRNA-seq Differentially Expressed Genes

File name: Supplementary Data 9
Description: Overlap of nucRNA-seq and ATAC-seq gene lists

File name: Supplementary Data 10
Description: nucRNA-seq Differentially Expressed Genes with an Egr1 motif in a differential ATAC-seq peak, Proestrus- Dioestrus comparison
