# Supplementary material for: Chromatin organization in the female mouse brain fluctuates across the oestrous cycle
Source: Nat Commun. 2019 Jun 28;10:2851. doi: 10.1038/s41467-019-10704-0 (PMC6598989; doi:10.1038/s41467-019-10704-0)
Supplement: Supplementary file 14 — Reporting Summary [file 41467_2019_10704_MOESM14_ESM.pdf]

nature research

Corresponding author(s): Marija Kundakovic

Reporting Summary

Nature Research wishes to improve the reproducibility of the work that we publish. This form provides structure for consistency and transparency in reporting. For further information on Nature Research policies, see Authors & Referees and the Editorial Policy Checklist.

Statistical parameters

When statistical analyses are reported, confirm that the following items are present in the relevant location (e.g. figure legend, table legend, main text, or Methods section).

n/a	Confirmed	
☐	☒	The exact sample size (*n*) for each experimental group/condition, given as a discrete number and unit of measurement
☐	☒	An indication of whether measurements were taken from distinct samples or whether the same sample was measured repeatedly
☐	☒	The statistical test(s) used AND whether they are one- or two-sided<br>*Only common tests should be described solely by name; describe more complex techniques in the Methods section.*
☒	☐	A description of all covariates tested
☐	☒	A description of any assumptions or corrections, such as tests of normality and adjustment for multiple comparisons
☐	☒	A full description of the statistics including central tendency (e.g. means) or other basic estimates (e.g. regression coefficient) AND variation (e.g. standard deviation) or associated estimates of uncertainty (e.g. confidence intervals)
☐	☒	For null hypothesis testing, the test statistic (e.g. *F*, *t*, *r*) with confidence intervals, effect sizes, degrees of freedom and *P* value noted<br>*Give P values as exact values whenever suitable.*
☒	☐	For Bayesian analysis, information on the choice of priors and Markov chain Monte Carlo settings
☒	☐	For hierarchical and complex designs, identification of the appropriate level for tests and full reporting of outcomes
☒	☐	Estimates of effect sizes (e.g. Cohen's *d*, Pearson's *r*), indicating how they were calculated
☐	☒	Clearly defined error bars<br>*State explicitly what error bars represent (e.g. SD, SE, CI)*

Our web collection on statistics for biologists may be useful.

Software and code

Policy information about availability of computer code

Data collection	Behavioural data were collected using the ANY-maze Video Tracking Software (ANY-maze 5.1, Stoelting Co; IL). Real-time qRT-PCR data were collected using QuantStudio™ Design and Analysis Desktop Software version 1.4 (Applied Biosystems; Thermo Fisher Scientific, Waltham, MA). Immunofluorescence data were processed using Leica confocal LAS AF software (Leica Microsystems GmbH). Golgi staining (spine density measurements) data were processed using Axiovision 4.8 Software (Carl Zeiss International). Flow cytometry data were collected using BD FACSDiva v8.0.1 software.
Data analysis	Behavioural data were initially analyzed by the ANY-maze Video Tracking Software (ANY-maze 5.1). Relative gene expression data were initially analyzed using QuantStudio™ Design and Analysis Desktop Software version 1.4. Statistical analysis of behavioral, gene expression, and hormone level data were performed using the IBM SPSS Statistics 25.0 software. Flow cytometry data were analyzed using BD FACSDiva v8.0.1 software.<br>We used the following software and packages for the bioinformatic analysis: picard-tools/1.92/java.1.8.0_20; bwa/0.7.15/gcc.4.4.7; samtools/1.5/gcc.4.4.7; FastQC/0.11.4/java.1.8.0_20; bedtools2/2.26.0/gcc.4.4.7; idr/2.0.2/python.3.4.1-atlas-3.11.30; MACS2/2.1.0-update/python.2.7.8; STAR/2.6.1b/gcc.4.9.2; R version 3.4.1; ChIPQC (https://www.bioconductor.org/packages/release/bioc/html/ChIPQC.html); DESeq2 (https://bioconductor.org/packages/release/bioc/html/DESeq2.html) ; clusterProfiler (https://bioconductor.org/packages/release/bioc/html/clusterProfiler.html); topGO (https://bioconductor.org/packages/release/bioc/html/topGO.html); g:Profiler (https://biit.cs.ut.ee/gprofiler/gost).<br>The code files are available at https://github.com/MasakoSuzuki/Jaric_etal_2018.<br>BAM files were visualized using Integrative Genomics Viewer - IGV version 2.3.92 (146). Genes showing differential chromatin accessibility in the KEGG serotonergic synapse pathway and ATAC-seq/nucRNA-seq overlap genes were visualized using STRING v10.5

(STRING Consortium 2017).
The number of dendritic spines was analyzed using ImageJ (public domain software from the National Institutes of Health; http://imagej.nih.gov/ij/).

For manuscripts utilizing custom algorithms or software that are central to the research but not yet described in published literature, software must be made available to editors/reviewers upon request. We strongly encourage code deposition in a community repository (e.g. GitHub). See the Nature Research guidelines for submitting code & software for further information.

Data

Policy information about availability of data

All manuscripts must include a data availability statement. This statement should provide the following information, where applicable:

- Accession codes, unique identifiers, or web links for publicly available datasets
- A list of figures that have associated raw data
- A description of any restrictions on data availability

ATAC-seq and nucRNA-seq data sets are available from the NCBI Gene Expression Omnibus (GEO) database under accession number GSE114036.

Field-specific reporting

Please select the best fit for your research. If you are not sure, read the appropriate sections before making your selection.

☒ Life sciences ☐ Behavioural & social sciences ☐ Ecological, evolutionary & environmental sciences

For a reference copy of the document with all sections, see nature.com/authors/policies/ReportingSummary-flat.pdf

Life sciences study design

All studies must disclose on these points even when the disclosure is negative.

Sample size	No statistical methods were used to predetermine sample size. Sample sizes were chosen based on our previous experience and published work (PMID:23716699, PMID:22955991). All behavioural tests included 12-16 animals per group. All whole-cell gene expression experiments included 8 animals per group. ATAC-seq and nucRNA-seq experiments included 3 biological replicates (each pooled from 2 animals) per group. Hormone level measurements included 6 (for serum) or 8 (for the hippocampus) animals per group. For validation of candidate genes revealed by the nucRNA-seq analysis, we used technical replicates of the same samples analyzed by nucRNA-seq. Histological experiments included 5 animals per group.
Data exclusions	Only one animal was excluded as an outlier (being more than 2 standard deviations from the group mean) from the analysis of elevated plus maze behavioural data, to avoid type II error. ATAC-seq data were down-sampled in order to reduce the peak number bias caused by unequal read numbers between the samples. The down-sampling factor was calculated based on the read numbers and RiP score (reads in peaks) from ChIPQC Bioconductor package (PMID: 24782889). Details are given in the Supplementary Table 1.
Replication	Since this was an animal study, we used biological replicates in all experiments (please see sample sizes). Behavioural data were also reproduced in two additional animal cohorts, which were ran independently, at different times.
Randomization	We were not able to do standard sample randomization since our experiments depended on natural hormone fluctuations in females. We allocated females to two different groups (proestrus or dioestrus) based on their natural cycling patterns and the exact day of testing depended on the duration of their oestrous cycles. However, we made sure to have equal group (proestrus, dioestrus, and males) distribution for each testing or in each batch (e.g. for sorting, library prep, sequencing), whenever possible.
Blinding	Blinding was not possible in this study since we used estrous cycle stage predictions for females and had to test animals at a particular stage of the oestrous cycle, which required careful planning and testing or tissue collection on a particular day and time of the day. For all experiments and sample processing that were done on different days, samples had to be equally distributed to control for batch effects. Bioinformaticians were aware of data generation, processing, and metadata.

Reporting for specific materials, systems and methods

Materials & experimental systems

n/a	Involved in the study
☐	☐ Unique biological materials
☐	☒ Antibodies
☐	☐ Eukaryotic cell lines
☐	☐ Palaeontology
☐	☒ Animals and other organisms
☐	☐ Human research participants

Methods

n/a	Involved in the study
☐	☒ ChIP-seq
☐	☒ Flow cytometry
☐	☐ MRI-based neuroimaging

Unique biological materials

Policy information about availability of materials

Obtaining unique materials	This study does not contain unique biological materials

Antibodies

Antibodies used	Antibodies for FACS: Mouse monoclonal Anti-NeuN Antibody, clone A60, conjugated to AlexaFluor 488 (MAB377X; Millipore); and Mouse monoclonal IgG1-k, clone MOPC-21 antibody control, conjugated to Alexa Fluor 488 (FCMAB310A4, Millipore).<br>Primary antibodies for immunofluorescence analysis: Rabbit polyclonal anti- ERβ antibody (Abcam, ab3576); Mouse monoclonal anti-DARPP32 antibody (Santa Cruz Biotechnology, sc-271111); Chicken anti-MAP2 antibody (Abcam, ab5392).<br>Secondary antibodies for immunofluorescence analysis: Anti-rabbit IgG Alexa Fluor 647 conjugate for ERβ visualization (Abcam, ab150079); Anti-mouse IgG CruzFluor 594 conjugate for DARPP32 visualization (Santa Cruz Biotechnology, sc-516178); Anti-chicken IgY Alexa Fluor 488 conjugate for MAP2 visualization (Abcam, ab150169).
Validation	Mouse monoclonal anti-NeuN antibody conjugated to AlexaFluor 488 (MAB377X; Millipore) - previously validated for fluorescence-activated nuclei sorting (PMID:27113501) and confirmed by immunofluorescence in this study.<br>Mouse monoclonal IgG1-k, clone MOPC-21 antibody control, conjugated to Alexa Fluor 488 (FCMAB310A4, Millipore) - validated for use in Flow Cytometry by the manufacturer.<br>Rabbit polyclonal anti- ERβ antibody (Abcam, ab3576): Validated by the manufacturer using WB on rat brain tissue homogenate and by IHC using cells over-expressing the human ERβ protein . Applications: IHC-P, IHC-Fr, ICC, WB, Inhibition Assay, ICC/IF. Species reactivity: mouse, rat, sheep, human, pig, non-human primates.<br>Mouse monoclonal anti-DARPP32 antibody (Santa Cruz Biotechnology, sc-271111): Validated by Western blot and IHC by manufacturer. Applications: WB, IP, IF, IHC(P) and ELISA. Species reactivity: mouse, rat, human and avian.<br>Chicken polyclonal anti-MAP2 antibody (Abcam, ab5392). Validated by WB, IHC, IF by manufacturer. Species reactivity: Mouse, Rat, Sheep, Cow, Dog, Human, Cynomolgus monkey, Common marmoset, Aplysia. Applications: ELISA, IHC-Fr, IHC-FoFr, IHC-P, WB, ICC/IF, IHC (PFA fixed).<br>Goat Anti-Rabbit IgG H&L (Alexa Fluor® 647) ab150079; Validated by manufacturer. Ex: 652nm, Em: 668nm; Applications: IHC-Fr, ICC/IF, ELISA, IHC-P, Flow Cyt.<br>Mouse IgG kappa binding protein (m-IgGκ BP) conjugated to CruzFluor™ 594 (CFL 594): sc-516178; Validated by manufacturer: Ex:592 nm, Em: 614 nm; Applications: IF, Flow Cyt.<br>Goat Anti-Chicken IgY H&L (Alexa Fluor® 488) (ab150169); Validated by manufacturer; Ex: 495nm, Em: 519nm; Applications: IHC-Fr, ICC/IF, ELISA, IHC-P, Flow Cyt.

Eukaryotic cell lines

Policy information about cell lines

Cell line source(s)	This study did not contain cell lines
Authentication	*Describe the authentication procedures for each cell line used OR declare that none of the cell lines used were authenticated.*
Mycoplasma contamination	*Confirm that all cell lines tested negative for mycoplasma contamination OR describe the results of the testing for mycoplasma contamination OR declare that the cell lines were not tested for mycoplasma contamination.*
Commonly misidentified lines (See ICLAC register)	*Name any commonly misidentified cell lines used in the study and provide a rationale for their use.*

Palaeontology

Specimen provenance	This study did not include fossil specimens
Specimen deposition	*Indicate where the specimens have been deposited to permit free access by other researchers.*
Dating methods	*If new dates are provided, describe how they were obtained (e.g. collection, storage, sample pretreatment and measurement), where they were obtained (i.e. lab name), the calibration program and the protocol for quality assurance OR state that no new*

dates are provided.

☐ Tick this box to confirm that the raw and calibrated dates are available in the paper or in Supplementary Information.

Animals and other organisms

Policy information about studies involving animals; ARRIVE guidelines recommended for reporting animal research

Laboratory animals	Male and female C57BL/6J mice were used in this study. Mice were obtained at 4 weeks of age from the Jackson Laboratory. Habituation took place from 4-6 weeks of age. The oestrous cycle in female animals was monitored daily for the duration of 3 oestrous cycles (6-8 weeks of age). At 8 weeks of age, animals were assigned to three different groups (proestrus females, diestrus females, and males) and underwent behavioural testing (8-10 weeks of age). Animals were sacrificed for molecular, hormone level, and histological analyses at 11 weeks of age. All animal procedures were approved by the Institutional Animal Care and Use Committee at Fordham University.
Wild animals	This study did not include wild animals
Field-collected samples	This study did not include field-collected samples

Human research participants

Policy information about studies involving human research participants

Population characteristics	This study did not include human subjects
Recruitment	*Describe how participants were recruited. Outline any potential self-selection bias or other biases that may be present and how these are likely to impact results.*

ChIP-seq

Data deposition

☒ Confirm that both raw and final processed data have been deposited in a public database such as GEO.

☒ Confirm that you have deposited or provided access to graph files (e.g. BED files) for the called peaks.

Data access links
May remain private before publication.

GEO accession GSE114036:
https://www.ncbi.nlm.nih.gov/geo/query/acc.cgi?acc=GSE114036

Files in database submission

For ATAC-seq:
Di_1_peaks.narrowPeak
Di_2_peaks.narrowPeak
Di_3_peaks.narrowPeak
Ma_1_peaks.narrowPeak
Ma_2_peaks.narrowPeak
Ma_3_peaks.narrowPeak
Pr_1_peaks.narrowPeak
Pr_2_peaks.narrowPeak
Pr_3_peaks.narrowPeak
Di_1.1_val_1.fq.gz
Di_1.2_val_2.fq.gz
Di_2.1_val_1.fq.gz
Di_2.2_val_2.fq.gz
Di_3.1_val_1.fq.gz
Di_3.2_val_2.fq.gz
Ma_1.1_val_1.fq.gz
Ma_1.2_val_2.fq.gz
Ma_2.1_val_1.fq.gz
Ma_2.2_val_2.fq.gz
Ma_3.1_val_1.fq.gz
Ma_3.2_val_2.fq.gz
Pr_1.1_val_1.fq.gz
Pr_1.2_val_2.fq.gz
Pr_2.1_val_1.fq.gz
Pr_2.2_val_2.fq.gz
Pr_3.1_val_1.fq.gz
Pr_3.2_val_2.fq.gz

For nucRNA-seq:
NRNA-1_CGATGT-_AHHNKMBCX2_L001_001.R1.fastq.gz
NRNA-1_CGATGT-_AHHNKMBCX2_L001_001.R2.fastq.gz
NRNA-1_CGATGT-_AHHNKMBCX2_L002_001.R1.fastq.gz
NRNA-1_CGATGT-_AHHNKMBCX2_L002_001.R2.fastq.gz

NRNA-2_CAGATC-_AHHNKMBCX2_L001_001.R1.fastq.gz
NRNA-2_CAGATC-_AHHNKMBCX2_L001_001.R2.fastq.gz
NRNA-2_CAGATC-_AHHNKMBCX2_L002_001.R1.fastq.gz
NRNA-2_CAGATC-_AHHNKMBCX2_L002_001.R2.fastq.gz
NRNA-3_GTGAAACG-_AHHNKMBCX2_L001_001.R1.fastq.gz
NRNA-3_GTGAAACG-_AHHNKMBCX2_L001_001.R2.fastq.gz
NRNA-3_GTGAAACG-_AHHNKMBCX2_L002_001.R1.fastq.gz
NRNA-3_GTGAAACG-_AHHNKMBCX2_L002_001.R2.fastq.gz
NRNA-4_ACAGTG-_AHHNKMBCX2_L001_001.R1.fastq.gz
NRNA-4_ACAGTG-_AHHNKMBCX2_L001_001.R2.fastq.gz
NRNA-4_ACAGTG-_AHHNKMBCX2_L002_001.R1.fastq.gz
NRNA-4_ACAGTG-_AHHNKMBCX2_L002_001.R2.fastq.gz
NRNA-5_GCCAAT-_AHHNKMBCX2_L001_001.R1.fastq.gz
NRNA-5_GCCAAT-_AHHNKMBCX2_L001_001.R2.fastq.gz
NRNA-5_GCCAAT-_AHHNKMBCX2_L002_001.R1.fastq.gz
NRNA-5_GCCAAT-_AHHNKMBCX2_L002_001.R2.fastq.gz
NRNA-6_ATGTCAGA-_AHHNKMBCX2_L001_001.R1.fastq.gz
NRNA-6_ATGTCAGA-_AHHNKMBCX2_L001_001.R2.fastq.gz
NRNA-6_ATGTCAGA-_AHHNKMBCX2_L002_001.R1.fastq.gz
NRNA-6_ATGTCAGA-_AHHNKMBCX2_L002_001.R2.fastq.gz
NRNA-7_TGACCA-_AHHNKMBCX2_L001_001.R1.fastq.gz
NRNA-7_TGACCA-_AHHNKMBCX2_L001_001.R2.fastq.gz
NRNA-7_TGACCA-_AHHNKMBCX2_L002_001.R1.fastq.gz
NRNA-7_TGACCA-_AHHNKMBCX2_L002_001.R2.fastq.gz
NRNA-8_CTTGTA-_AHHNKMBCX2_L001_001.R1.fastq.gz
NRNA-8_CTTGTA-_AHHNKMBCX2_L001_001.R2.fastq.gz
NRNA-8_CTTGTA-_AHHNKMBCX2_L002_001.R1.fastq.gz
NRNA-8_CTTGTA-_AHHNKMBCX2_L002_001.R2.fastq.gz
NRNA-9_CCGTCCCG-_AHHNKMBCX2_L001_001.R1.fastq.gz
NRNA-9_CCGTCCCG-_AHHNKMBCX2_L001_001.R2.fastq.gz
NRNA-9_CCGTCCCG-_AHHNKMBCX2_L002_001.R1.fastq.gz
NRNA-9_CCGTCCCG-_AHHNKMBCX2_L002_001.R2.fastq.gz
NRNA-1_CGATGT-_AHHNKMBCX2ReadsPerGene.out.tab
NRNA-2_CAGATC-_AHHNKMBCX2ReadsPerGene.out.tab
NRNA-3_GTGAAACG-_AHHNKMBCX2ReadsPerGene.out.tab
NRNA-4_ACAGTG-_AHHNKMBCX2ReadsPerGene.out.tab
NRNA-5_GCCAAT-_AHHNKMBCX2ReadsPerGene.out.tab
NRNA-6_ATGTCAGA-_AHHNKMBCX2ReadsPerGene.out.tab
NRNA-7_TGACCA-_AHHNKMBCX2ReadsPerGene.out.tab
NRNA-8_CTTGTA-_AHHNKMBCX2ReadsPerGene.out.tab
NRNA-9_CCGTCCCG-_AHHNKMBCX2ReadsPerGene.out.tab

Genome browser session
(e.g. UCSC)

Provide a link to an anonymized genome browser session for "Initial submission" and "Revised version" documents only, to enable peer review. Write "no longer applicable" for "Final submission" documents.

Methodology

Replicates

For ATAC-seq experiments, three biological replicates (each pooled from two animals) were analyzed per group. 6 animals per group were initially processed individually, including nuclei preparation, sorting, library prep, and sequencing. Each pair of samples belonging to the same group (proestrus, dioestrus, or males) and sorting batch (we had 3 sorting batches with equal group distribution, n=2/group) were merged for the bionformatic analysis (resulting in n=3 per group). This was performed to reduce technical variability and to obtain the data with optimal quality control (FRiP) scores. For nucRNA-seq experiments, three biological replicates (each pooled from two animals) were analyzed per group. The pooling for these experiments was performed immediately after ventral hippocampal dissection so that we had three biological samples per group throughout the procedure, from nuclei preparation to sequencing.

Sequencing depth

For ATAC-seq, we performed 100 bp paired-end sequencing using the Illumina HiSeq 2500 instrument. We obtained 86 to 132 million reads per sample (mean=108,382,828 and standard deviation= 14,003,920). Detailed information is listed in the Suppl. Table 1.
For nucRNA-seq, we performed 100 bp paired-end sequencing using the Illumina HiSeq 4000 instrument. We obtained 39 to 42 million paired reads per sample (mean=41,290,882 and standard deviation=1,428,555). Detailed information is listed in the Suppl. Table 2.

Antibodies

We performed ATAC-seq and nucRNA-seq experiments and, therefore, the antibodies were not used

Peak calling parameters

Peak calling for ATAC-seq:
1. Alignment
Alignment was performed using BWA mem package (bwa/0.7.15/gcc.4.4.7) and only uniquely aligned reads were used in the analysis. Alignment.sh has the code used in this study. The chr.bed is the list of each canonical chromosome length.
2. Peak-calling
Peak-calling was performed following the original ATAC-seq protocol (PMID:24097267) using macs2 (MACS2/2.1.0-update/python.2.7.8) on the shifted read1. Peak_call.sh has the code used in this study.
3. Down-sampling
Based on the number of reads in peaks (NPR), we calculated down-sampling factor. Down_sampling.sh has the code used in this study.

Down-sampling factor = NRP of the sample / mean NRP of all samples
Note: if the factor is greater than 1, we used one as the down-sampling factor.
4. Second round peak-calling
We re-performed peak-calling on the down-sampled bam files using the same parameters as described in step 2.
Peak_call.sh has the code used in this study.
5. Irreproducible Discovery Rate (IDR)
IDR was calculated with idr (idr/2.0.2/python.3.4.1-atlas-3.11.30). The obtained peaks were merged with bedtools merge function (bedtools2/2.26.0/gcc.4.4.7). We used 0.05 as the threshold. IDR_cal.sh has the code used in this study.

Data quality

For ATAC-seq, we performed QC on the obtained reads with the ChIPQC Bioconductor package. The stats were listed in Suppl. Table 1 in the manuscript. We obtained 180,589 to 310,212 peaks at FDR<0.05 (average=229,639, standard deviation=44,830). QC_analysis.rmd has the code used in this study.
For nucRNA-seq, we assessed the sequence quality by FastQC and the library quality by RSeQC. The stats were listed in Suppl. Table 2 in the manuscript.

Software

We used the following software and packages:
picard-tools/1.92/java.1.8.0_20
bwa/0.7.15/gcc.4.4.7
samtools/1.5/gcc.4.4.7
FastQC/0.11.4/java.1.8.0_20
bedtools2/2.26.0/gcc.4.4.7
idr/2.0.2/python.3.4.1-atlas-3.11.30
MACS2/2.1.0-update/python.2.7.8
STAR/2.6.1b/gcc.4.9.2
R version 3.4.1
ChIPQC (https://www.bioconductor.org/packages/release/bioc/html/ChIPQC.html)
DESeq2 (https://bioconductor.org/packages/release/bioc/html/DESeq2.html)
clusterProfiler (https://bioconductor.org/packages/release/bioc/html/clusterProfiler.html)
topGO (https://bioconductor.org/packages/release/bioc/html/topGO.html)
g:Profiler (https://biit.cs.ut.ee/gprofiler/gost)

The code files are available at https://github.com/MasakoSuzuki/Jaric_etal_2018

Flow Cytometry

Plots

Confirm that:

☒ The axis labels state the marker and fluorochrome used (e.g. CD4-FITC).

☒ The axis scales are clearly visible. Include numbers along axes only for bottom left plot of group (a 'group' is an analysis of identical markers).

☒ All plots are contour plots with outliers or pseudocolor plots.

☒ A numerical value for number of cells or percentage (with statistics) is provided.

Methodology

Sample preparation

Ventral hippocampus was dissected from the brain, snap frozen in liquid nitrogen, and total nuclei were extracted using sucrose gradient centrifugation. Neuronal (NeuN+) nuclei were isolated by fluorescence-activated nuclei sorting with anti-NeuN antibody conjugated to AlexaFluor 488 on a FACSAria instrument. Detailed protocol for total nuclei isolation and purification of neuronal nuclei is provided in Kundakovic et al, 2017 (PMID:27113501)

Instrument

FACS Aria instrument (BD Sciences, San Jose, CA); Cat No: 337667

Software

BD FACSDiva v8.0.1 software

Cell population abundance

For ATAC-seq, 30, 000 neuronal nuclei from mouse ventral hippocampus were collected per each sample. For ATAC-seq, 250,000 neuronal nuclei from mouse ventral hippocampus were collected per each sample.

Gating strategy

To set up the experimental protocol for fluorescent activated nuclei sorting on a FACSAria instrument, we used three controls: i) DAPI only; ii) IgG1 isotype control-AlexaFluor 488 and DAPI; and iii) NeuN-AlexaFluor 488 only; in addition to a sample containing NeuN-AlexaFluor 488 and DAPI stain. For each sample, gates were adjusted to: i) select nuclei from debris; ii) ensure single nuclear sorting (using DAPI); and iii) select the NeuN+ (neuronal) and NeuN- (non-neuronal) nuclei populations. The gating strategy is presented in Supplementary Figure 5. We have previously described this method in great detail (PMID:27113501).

☒ Tick this box to confirm that a figure exemplifying the gating strategy is provided in the Supplementary Information.

Magnetic resonance imaging

Experimental design

Design type

This study did not include magnetic resonance imaging

Design specifications	*Specify the number of blocks, trials or experimental units per session and/or subject, and specify the length of each trial or block (if trials are blocked) and interval between trials.*
Behavioral performance measures	*State number and/or type of variables recorded (e.g. correct button press, response time) and what statistics were used to establish that the subjects were performing the task as expected (e.g. mean, range, and/or standard deviation across subjects).*

Acquisition

Imaging type(s)	*Specify: functional, structural, diffusion, perfusion.*
Field strength	*Specify in Tesla*
Sequence & imaging parameters	*Specify the pulse sequence type (gradient echo, spin echo, etc.), imaging type (EPI, spiral, etc.), field of view, matrix size, slice thickness, orientation and TE/TR/flip angle.*
Area of acquisition	*State whether a whole brain scan was used OR define the area of acquisition, describing how the region was determined.*

Diffusion MRI ☐ Used ☒ Not used

Preprocessing

Preprocessing software	*Provide detail on software version and revision number and on specific parameters (model/functions, brain extraction, segmentation, smoothing kernel size, etc.).*
Normalization	*If data were normalized/standardized, describe the approach(es): specify linear or non-linear and define image types used for transformation OR indicate that data were not normalized and explain rationale for lack of normalization.*
Normalization template	*Describe the template used for normalization/transformation, specifying subject space or group standardized space (e.g. original Talairach, MNI305, ICBM152) OR indicate that the data were not normalized.*
Noise and artifact removal	*Describe your procedure(s) for artifact and structured noise removal, specifying motion parameters, tissue signals and physiological signals (heart rate, respiration).*
Volume censoring	*Define your software and/or method and criteria for volume censoring, and state the extent of such censoring.*

Statistical modeling & inference

Model type and settings	*Specify type (mass univariate, multivariate, RSA, predictive, etc.) and describe essential details of the model at the first and second levels (e.g. fixed, random or mixed effects; drift or auto-correlation).*
Effect(s) tested	*Define precise effect in terms of the task or stimulus conditions instead of psychological concepts and indicate whether ANOVA or factorial designs were used.*

Specify type of analysis: ☐ Whole brain ☐ ROI-based ☐ Both

Statistic type for inference (See Eklund et al. 2016)	*Specify voxel-wise or cluster-wise and report all relevant parameters for cluster-wise methods.*
Correction	*Describe the type of correction and how it is obtained for multiple comparisons (e.g. FWE, FDR, permutation or Monte Carlo).*

Models & analysis

n/a	Involved in the study
☒	☐ Functional and/or effective connectivity
☒	☐ Graph analysis
☒	☐ Multivariate modeling or predictive analysis
